# The Presence of Thyroid-Stimulation Blocking Antibody Prevents High Bone Turnover in Untreated Premenopausal Patients with Graves’ Disease

**DOI:** 10.1371/journal.pone.0144599

**Published:** 2015-12-09

**Authors:** Sun Wook Cho, Jae Hyun Bae, Gyeong Woon Noh, Ye An Kim, Min Kyong Moon, Kyoung Un Park, Junghan Song, Ka Hee Yi, Do Joon Park, June-Key Chung, Bo Youn Cho, Young Joo Park

**Affiliations:** 1 Department of Internal Medicine, Seoul National University College of Medicine, Seoul, South Korea; 2 Department of Nuclear Medicine, Seoul National University Hospital, Seoul, South Korea; 3 Department of Internal Medicine, Boramae Medical Center, Seoul, South Korea; 4 Department of Laboratory Medicine, Seoul National University Bundang Hospital, Seongnam, South Korea; 5 Department of Internal Medicine, Chung-Ang University College of Medicine, Seoul, South Korea; Kyungpook National University School of Medicine, REPUBLIC OF KOREA

## Abstract

Osteoporosis-related fractures are one of the complications of Graves’ disease. This study hypothesized that the different actions of thyroid-stimulating hormone receptor (TSHR) antibodies, both stimulating and blocking activities in Graves’ disease patients might oppositely impact bone turnover. Newly diagnosed premenopausal Graves’ disease patients were enrolled (n = 93) and divided into two groups: patients with TSHR antibodies with thyroid-stimulating activity (stimulating activity group, n = 83) and patients with TSHR antibodies with thyroid-stimulating activity combined with blocking activity (blocking activity group, n = 10). From the stimulating activity group, patients who had matched values for free T4 and TSH binding inhibitor immunoglobulin (TBII) to the blocking activity group were further classified as stimulating activity-matched control (n = 11). Bone turnover markers BS-ALP, Osteocalcin, and C-telopeptide were significantly lower in the blocking activity group than in the stimulating activity or stimulating activity-matched control groups. The TBII level showed positive correlations with BS-ALP and osteocalcin levels in the stimulating activity group, while it had a negative correlation with the osteocalcin level in the blocking activity group. In conclusion, the activation of TSHR antibody-activated TSH signaling contributes to high bone turnover, independent of the actions of thyroid hormone, and thyroid-stimulation blocking antibody has protective effects against bone metabolism in Graves’ disease.

## Introduction

Osteoporosis is an established complication of Graves’ disease, which is a common endocrine disorder affecting 1%–2% of individuals [1–3]. Several studies have shown that Graves’ disease patients exhibit low bone mineral density and increased risk of bone fracture [4]. Thyroid hormone has a pivotal role in metabolic changes of the bone in Graves’ disease patients because it stimulates both osteoclast [5] and osteoblast [6] activities, with the net effect being the negative regulation of bone mass [7–9].

Apart from the effects of the thyroid hormone, thyroid-stimulating hormone (TSH) signaling also has been suggested to have a role in bone metabolism. Although it is hard to define the significance of TSH action independent from thyroid hormone in bone metabolism, because TSH and thyroid hormone move in reciprocal directions within a hormonal feedback mechanism. Several studies on subclinical thyroid disease, in which status only TSH is elevated or decreased without a change of thyroid hormone, have shown that TSH levels had positive correlations with BMD [10–12]. Moreover, differentiated thyroid cancer patients who received therapeutic higher doses of thyroid hormone replacement, resulting in factitious subclinical hyperthyroidism, showed an increase risk of osteoporosis [13–14]. A study from peri-menopausal Dutch women showed that higher thyroxine levels within the normal reference range, but not lower or undetectable serum TSH levels, were independently associated with low BMD [15], indicating that thyroid hormone rather than TSH had pivotal roles in bone metabolism.

On the other hands, TSH receptor antibodies (TSHR antibodies) can activate TSH receptor similar to TSH in thyrocytes (16). In Graves’ disease, although TSH is markedly suppressed, the TSHR antibodies present in the patients’ sera, thus it could stimulate the TSH signaling of cells. Moreover, after reaching the remission of Graves’ disease, TSHR antibodies could exist persistently in their sera, especially when treated with radioactive iodine [16–17]. Thus, the effect of TSHR signaling on bone metabolism is of interest to predict long term clinical outcomes of their bone health.

Given that TSHR signaling has a functional role in the bone metabolism of hyperthyroidism, we hypothesized that the different actions of TSHR antibodies, both stimulating and blocking activities in Graves’ disease patients, might oppositely impact bone metabolism. The relationship between TSHR antibodies and serum bone turnover markers were analyzed in untreated premenopausal patients with Graves’ disease.

## Subjects and Methods

### Subjects

Newly diagnosed Graves’ disease patients who were treated at Seoul National University Hospital between March 2001 and December 2001 were screened, and 93 premenopausal women (mean age, 33±9 years) were enrolled. The presence of Graves’ disease was diagnosed based on symptoms and signs of hyperthyroidism including high thyroxine (T4) and/or triiodothyronine (T3) concentrations, suppressed TSH levels, and markedly increased uptake (>4.5%) on technetium-99m thyroid scans. The enrolled patients had no history of previous fractures, intake of supplements or drugs such as calcium, vitamin D, or steroids that could affect bone metabolism, or any other autoimmune diseases. The study was approved by the institutional review board of Seoul National University Hospital (IRB 1204-060-406). Verbal informed consent was obtained from each patient, and samples were collected and used with encryption. All participants were provided an oral statement about the research including the basic elements of the informed consent. Because this study was performed before the establishment of the ‘Bioethics and Safety Act’ in Korea (2005.3.24), the IRB approved a waiver of documentation for consent.

### Classification of ‘stimulating activity’ and ‘blocking activity’ groups

All 93 patients were divided into two groups according to their types of TSHR antibodies. Patients who had thyroid-stimulating activity alone were called the ‘stimulating activity’ group (n = 83). On the other hand, 10 patients who were clinically suspected to have both the thyroid stimulating antibody and thyroid-stimulation blocking antibody were called the ‘blocking activity’ group (n = 10). These 10 patients in the blocking activity group had spontaneous hypothyroidism with a persistent TSH binding inhibitor immunoglobulin (TBII) titer at 1–2 years after initial remission (S1 Table). Additionally, to further exclude the effects of the thyroid hormone and TBII titer on bone metabolism, 11 patients from the stimulating activity group who had matched values for the initial free T4 and TBII to those 10 patients in the blocking activity group were classified as the ‘stimulating activity-matched control’ group (n = 11). S1 Fig shows that the blocking activity group had an early response to anti-thyroid drug treatment (S1A Fig) compared to the stimulating activity-matched control group (S1B Fig).

### cAMP assay in normal thyroid epithelial cells

To validate the biological actions of the blocking activity, normal thyroid epithelial cells, H tori, which were purchased from the Korean cell line bank (Seoul, South Korea) and cultured in RPMI containing 10% fetal bovine serum, were treated with whole sera from individual patients in the blocking activity group (#1–#10). Recombinant bovine TSH (10 mUI/ml, Sigma-Aldrich, St. Louis, MO, USA), TSHR-stimulating human monoclonal antibody M22 (100 ng/mL, RSR Ltd., Cardiff, UK) and whole sera from two individual patients (#36 and #84) from the stimulating activity-matched control were used as positive controls in H tori cells, and TSHR-blocking human monoclonal autoantibody K1-70 (100 ng/mL, RSR Ltd.) was used as a negative control in H tori cells. After 1 h, proteins were harvested, and cAMP assays (R&D Systems, Minneapolis, MN, USA) were performed according to the manufacturer’s instructions.

### Biochemistry

Serum samples were obtained in the morning after overnight fasting and immediately processed and frozen at −70°C until the assays were done. The levels of serum TSH were measured by immunoradiometric assay (Daiichi Radioisotope Laboratories, Tokyo, Japan). The serum free T_4_ and T_3_ levels were measured by radioimmunoassay (Dainabot Radioisotope Laboratories, Tokyo, Japan). The levels of the TSHR antibody were measured using first generation TBII assay (RSR Ltd., Cardiff, UK). Serum concentrations of calcium, phosphorous, total protein, and albumin were measured on a Hitachi 7600 chemistry autoanalyzer (Hitachi, Tokyo, Japan). The levels of serum corrected calcium were calculated with the following equation; corrected calcium = 0.8 × (4.1—patients’ albumin) + patient’s calcium. Serum parathyroid hormone (PTH) levels were measured by immunoradiometric assay (Cisbio Bioassays, Parc Marcel Boiteux, Codolet, France), and serum 25-hydroxyvitamin D3 (25-OHD3) levels were measured with the Diels-Alder derivatization and ultraperformance liquid chromatography-tandem mass spectrometry (Waters, Milford, MA, USA). The levels of serum bone-specific alkaline phosphatase (BS-ALP) were measured by enzyme immunoassay (reference range of premenopausal women: 11.6–29.6 U/L, Quidel, San Diego, CA, USA). Serum osteocalcin levels were measured by immunoradiometric assay (Cisbio Bioassays, Parc Marcel Boiteux, Codolet, France). Serum C-terminal telopeptide of type I collagen (C-telopeptide) levels were measured with the electrochemiluminescence immunoassay (reference range of premenopausal women: <0.573 ng/mL, Roche, Indianapolis, IN, USA).

### Statistical analyses

Data are expressed as the mean±SD, and inter group differences were assessed with the Student’s *t*-test. Partial correlation coefficient results were calculated with SPSS 20.0 (SPSS, Chicago, IL, USA). A *P*-value < 0.05 was considered statistically significant.

## Results

### Comparisons of biochemical parameters related to thyroid and bone metabolism between the stimulating activity and blocking activity groups

Basal clinical and biochemical characteristics of the enrolled patients are summarized in Table 1. The mean diagnostic age was 33 ± 9 years; the serum free T4 level was 3.1 ± 1.8 ng/dL, and the mean TBII activity was 54.5 ± 24.8%. To explore the effects of the TSHR antibodies on bone metabolism, patients were divided into two groups based on the different activities of the TSHR antibodies: stimulating activity and blocking activity groups. The blocking activity of the TSHR antibody was verified by cAMP assay in thyrocytes using the whole sera of individual patients. Blocking activity group showed lower levels of cAMP production than that of the stimulating activity-matched control group (4.8±0.3 vs 12.9±0.5, P = 0.001), while it was higher than that of the monoclonal blocking antibody, K1-70 (4.8±0.3 vs 2.6±0.3, P<0.001). The mean diagnostic age and TBII activity level were higher, while the mean free T4 level was lower in the blocking activity group than in the stimulating activity group (Table 1). Among the bone-related parameters, calcium, phosphorus, 25-OHD3, and PTH levels were within their normal ranges in both the stimulating activity and blocking activity groups, and no significant differences between the two groups were detected (Table 1). However compared to the reference range, the level of bone formation marker BS-ALP was higher in all of our Graves’ disease patients. Moreover the BS-ALP level was significantly higher in the stimulating activity group than in the blocking activity group (58.2 ± 27.2 *vs*. 41.7 ± 15.8, *P* = 0.034; Table 1). Osteocalcin and C-telopeptide levels were above the reference ranges in the stimulating activity group but not in the blocking activity group.

**Table 1:** Clinical characteristics and serum parameters related with bone metabolism of enrolled patients.

	Normal reference range for premenopausal women	Stimulating activity		Blocking activity (n = 10)	^a^ *P*-value	^b^ *P*-value
		Total (n = 83)	Matched control (n = 11)			
Age (years)		32 ± 9	38 ± 11	43 ± 13	0.033	0.351
Free T4 (ng/dL)		3.4 ± 1.5	2.6 ± 0.3	2.4 ± 0.3	<0.001	0.233
TBII (%)		53.7 ± 23.5	63.3 ± 8.6	74.9 ± 21.9	0.001	0.270
Calcium (mg/dL)		9.8 ± 0.4	9.8 ± 0.3	9.6 ± 0.8	0.790	0.669
Phosphorous (mg/dL)		4.4 ± 1.0	4.8 ± 1.2	4.2 ± 1.2	0.628	0.885
25-OHD3 (ng/mL)		16.7 ± 8.7	20.6 ± 9.7	18.3 ± 4.6	0.667	0.573
PTH (pg/mL)		12.8 ± 7.9	13.0 ± 4.6	13.9 ± 7.9	0.774	0.521
BS-ALP (U/L)	11.6–29.6	58.2 ± 27.2	65.0 ± 23.4	41.7 ± 15.8	0.034	0.006
Osteocalcin (ng/mL)	12–41	46.6 ± 19.6	49.4 ± 21.3	31.8 ± 18.5	0.025	0.049
C-telopeptide (ng/mL)	< 0.573	0.78 ± 0.35	0.81 ± 0.35	0.38 ± 0.37	0.001	0.013

^a^ *P*-value is from t-test between stimulating activity and blocking activity groups.

^b^ *P*-value is from t-test between stimulating activity-matched control and blocking activity groups.

Stimulating activity, patients with thyroid-stimulating activity alone; blocking activity, patients with thyroid-stimulating activity combined with blocking activity; stimulating activity-matched control, patients from stimulating activity group who had matched values of initial free T4 and TBII to blocking activity group; TBII, TSH binding inhibitor immunoglobulin; 25-OHD3, 25-hydroxyvitamin D3; PTH, parathyroid hormone; BS-ALP, bone-specific alkaline phosphatase.

Next, to exclude the effects of the thyroid hormone and TBII titer on bone metabolism, we then selected 11 patients from the stimulating activity group who had matched values for free T4 and TBII to those 10 patients in the blocking activity group and classified them as ‘stimulating activity- matched control’. Interestingly, serum bone turnover markers such as BS-ALP, osteocalcin, and C-telopeptide were significantly higher in the stimulating activity-matched control group than in the blocking activity group (Table 1). Taken together, the results indicate that the stimulating activity group included patients with high bone turnover phenotypes, and such phenotypes were less common in the blocking activity group.

### Association between TSH receptor antibody activity and bone turnover markers

**Fig 1:**
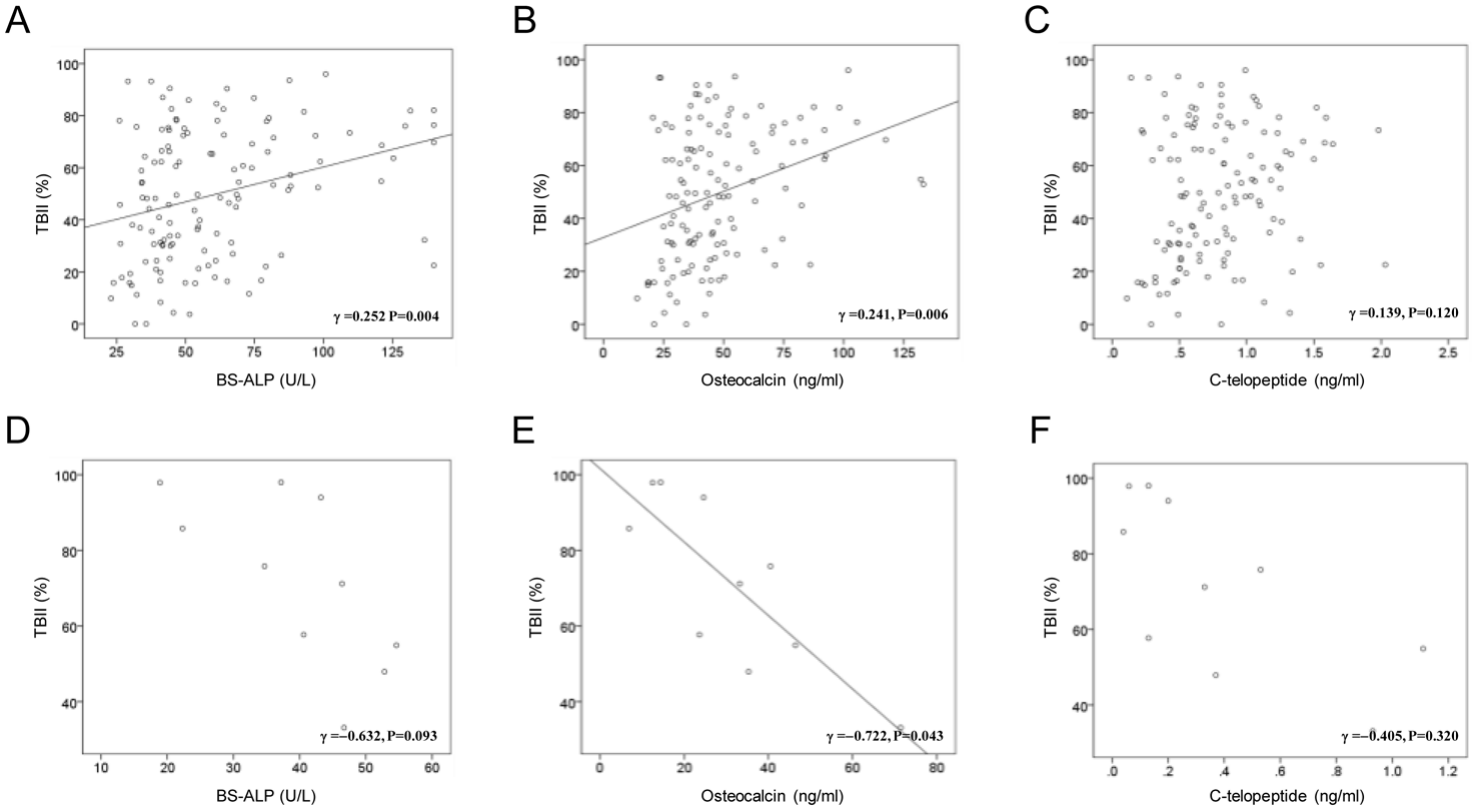
Correlations between TSHR antibodies and bone turnover markers. Correlations between TSH binding inhibitor immunoglobulin (TBII) levels (%) and bone turnover marker levels including bone-specific alkaline phosphatase (BS-ALP), osteocalcin, and C-telopeptide in stimulating activity (A, B, and C, respectively) and blocking activity groups (D, E, and F, respectively). Stimulating activity, patients with TSHR antibodies with thyroid-stimulating activity; Blocking activity, patients with TSHR antibodies with thyroid-stimulating activity combined with blocking activity.

Next, the association between the TBII activity levels and bone turnover marker concentrations were explored. In the stimulating activity group, TBII activity was positively correlated with BS-ALP (γ = 0.252, *P =* 0.004, Fig 1A) and osteocalcin (γ = 0.241, *P =* 0.006, Fig 1B) levels after adjustments for age, T3, and free T4 levels. Serum C-telopeptide also showed trends of positive correlation with TBII in the stimulating activity group without any statistical significance (γ = 0.139, *P =* 0.120, Fig 1C). In contrast, in the blocking activity group, the TBII activity showed non-significant trends toward negative correlations with C-telopeptide (γ = −0.541, *P* = 0.086, Fig 1D), and a strong negative correlation with osteocalcin (γ = −0.722, *P* = 0.043, Fig 1E), but not with serum C-telopeptide (γ = −0.405, *P =* 0.320, Fig 1F). Taken together, these data suggest that TSHR signaling has a functional role in bone metabolism, and the presence of thyroid-stimulation blocking antibody negatively impacts bone turnover.

## Discussion

The present study evaluated the role of TSHR signaling on bone metabolism in untreated hyperthyroidism patients. Among the 93 patients, 10 patients had the thyroid-stimulation blocking antibody along with the thyroid stimulating antibody (blocking activity group), while the others had the thyroid stimulating antibody alone (stimulating activity group). The presence of thyroid-stimulation blocking antibody attenuated the increased bone turnover in hyperthyroidism. TBII showed a negative correlation with serum osteocalcin in the blocking activity group, although it showed positive correlations between serum BS-ALP and osteocalcin in the stimulating activity group. These data suggest that, independent of the effects of thyroid hormone, TSHR antibody-activated TSH signaling, *per se*, might play a role in bone metabolism in Graves’ disease. To our knowledge, this is the first indication of the opposing roles of the different types of TSHR antibodies in bone metabolism.

Whether TSHR antibodies have independent effects on bone metabolism is still unclear. Regarding the effects of TSH, Abe et al. raised the hypothesis that TSH has independent roles on bone remodeling. In their study, TSH inhibited both osteoclastogenesis and osteoblastogenesis, and TSHR knockout mice showed marked bone loss with a high remodeling skeletal phenotype, suggesting a negative role of TSH in bone metabolism [18]. However, similar actions of TSH resulted in different bone phenotypes according to different pathophysiologic statuses. In rodents, intermittent administration of TSH to ovariectomized mice or rats showed an anti-resorptive and anabolic response to acute estrogen withdrawal [19–20]. In hyperthyroidism status, mice lacking TSHR showed profound bone loss with increased bone resorption compared to wild type mice [21], suggesting that TSH had protective effects in hyperthyroid-associated osteoporosis. Collectively, these data indicate that TSH could attenuate the bone turnover rate by modulating both resorption and formation, which are not in agreement with the present study. One of the possible explanations is that the antibody-based blocking activity could have discordance with that of the genetically engineered receptor knock out models. Furthermore, the actions of TSH signaling might be different in various conditions such as estrogen withdrawal and direct comparisons between murine biological systems to that of humans also caused limitations.n the other hand, TSH has been shown to have trivial effect on osteoblast differentiation *in vitro* [22]. Hyt/*hyt* mice harboring loss of function mutation of *TSHR* showed normal skeletal development [22], indicating negligible role of TSH on bone growth. Although the impacts of TSHR signaling on bone metabolism are not comparable to that of thyroid hormone, it is still valuable to define the independent role of TSHR signaling on bone metabolism especially in hyperthyroidism. Osteoporosis and related clinical fracture is one of the major long-term complications in Graves’ disease [4,23,24]. A large population-based study demonstrated that adjusted odd ratio for hip fracture is still increased more than 10 years after the diagnosis of hyperthyroidism [3]. Considering that the large portions of these patients still have TSH stimulating antibodies after reaching the remission status, the activated TSHR signaling might play a role in hyperthyroidism-related bone disease. Indeed, it has been reported that TSH stimulating antibody levels are positively correlated with bone turnover marker levels and negatively correlated with bone mineral density [25–26].

The aim of the present study was to clarify the functional roles of antibody-mediated TSHR signaling in bone metabolism. We showed that two different types of TSHR antibodies have opposing effects on bone metabolism. Clinically, TSHR antibodies are divided into two types: thyroid-stimulating antibody, which mimics the action of TSH resulting in autoimmune hyperthyroidism including Graves’ disease, and thyroid-stimulation blocking antibody which blocks the binding affinity of TSH to TSHR resulting in autoimmune hypothyroidism [27–29]. Although thyroid-stimulation blocking antibodies are detected mainly in patients with autoimmune hypothyroidism, they have also been observed, in company with thyroid-stimulating antibodies, in a small number of Graves’ disease patients [30]. The presence of thyroid-stimulation blocking antibodies in a subgroup of Graves’ disease patients might be the reason for the severe fluctuation of thyroid hormone levels observed in that subgroup. The present study investigated a subgroup of Graves’ disease patients that was considered to have thyroid-stimulation blocking antibodies, based on the patients’ severe fluctuations in thyroid hormone levels and a clinical outcome of spontaneous hypothyroidism, and verified that these patents showed a relatively low bone turnover rate compared to that in other Graves’ disease patients. Moreover, among the blocking activity group, the TSHR antibody titer was negatively correlated with bone turnover marker levels in this subgroup. These results support the hypothesis that TSH signaling, *per se*, has a role in the alteration of bone metabolism in Graves’ disease. Unfortunately, the molecular characteristics of the thyroid-stimulation blocking antibodies were not validated by direct measurement in this study. Currently, there is no direct method which can measure the activities of thyroid-stimulation blocking antibodies separately from thyroid-stimulating antibodies. The *in vitro* conversion method using FRTL-5 cells had been used, but still, it is an indirect method [29,31]. Because thyroid-stimulation blocking antibodies might be used not only in predicting the clinical outcomes of Graves’ disease but also in presuming bone health, further studies are need to develop new bioassays for detecting coexisting thyroid-stimulation blocking antibodies in the abundance of thyroid-stimulating antibodies.

The key finding of the present study is that the thyroid-stimulation blocking antibody attenuated the increased bone turnover in hyperthyroidism. First, bone turnover markers such as serum BS-ALP, osteocalcin, and C-telopeptide were higher than the normal range in the stimulating activity group, while they were lower in the blocking activity group. Second, the blocking activity group showed negative correlations between TBII (%) and bone turnover markers, while the stimulating activity groups showed an opposite direction in the correlations. Although BS-ALP (γ = -0.632, P = 0.093) and C-telopeptide (γ = -0.405, P = 0.320) did not obtain any statistical significant, they showed negative trends. The lack of statistical significance might be due to the small sample size. These results are consistent with a recent German population-based cohort study which showed strong positive correlations between the TSH levels and bone turnover markers including N-terminal propeptide of type I procollagen, BS-ALP, osteocalcin, and C-telopeptide [32].

This study has some limitations in investigating the role of antibody-mediated TSHR signaling on bone metabolism. Because bone mass was not evaluated, we could not deduce the net effects of the TSHR antibodies on bone remodeling. Moreover, bone-related clinical characteristics such as body weight were not measured in this study. A prospective, well-designed study is needed to validate the exact actions of antibody-mediated TSHR signaling in Graves’ patients with bone disease.

In conclusion, our results suggest that the activation of TSHR antibody-activated TSH signaling contributes to high bone turnover, independent of the actions of the thyroid hormone, and thyroid-stimulation blocking antibody has protective effects on bone metabolism in Graves’ disease.

## Supporting Information

S1 Fig Changes of FT4 during initial 6 months of therapy of Graves’ disease. (DOCX)

S1 Table Clinical characteristics of individual patients with blocking activity group (n = 10). Changes of serum FT4 concentrations during initial 6 months of anti-thyroid drug therapies (black circle, methimazole; white circle, propylthiouracil) from representative patients were demonstrated. (A) Stimulating activity-matched control group, and (B) blocking activity group. Stimulating activity, patients with thyroid-stimulating activity alone; blocking activity, patients with thyroid-stimulating activity combined with blocking activity; stimulating activity-matched control, patients from stimulating activity group who had matched values of initial free T4 and TBII to blocking activity group.
(DOCX)
