# Supplementary material for: The Presence of Thyroid-Stimulation Blocking Antibody Prevents High Bone Turnover in Untreated Premenopausal Patients with Graves’ Disease
Source: PLoS One. 2015 Dec 9;10(12):e0144599. doi: 10.1371/journal.pone.0144599 (PMC4674124; doi:10.1371/journal.pone.0144599)
Supplement: S1 Fig — (DOCX) [file pone.0144599.s001.docx]

**S1 Fig. Changes of FT4 during initial 6 months of therapy of Graves’ disease.**

Changes of serum FT4 concentrations during initial 6 months of anti-thyroid drug therapies (black circle, methimazole; white circle, propylthiouracil) from representative patients were demonstrated. (A) Stimulating activity-matched control group, and (B) blocking activity group. Stimulating activity, patients with thyroid-stimulating activity alone; blocking activity, patients with thyroid-stimulating activity combined with blocking activity; stimulating activity-matched control, patients from stimulating activity group who had matched values of initial free T4 and TBII to blocking activity group.
