# Supplementary material for: The Presence of Thyroid-Stimulation Blocking Antibody Prevents High Bone Turnover in Untreated Premenopausal Patients with Graves’ Disease
Source: PLoS One. 2015 Dec 9;10(12):e0144599. doi: 10.1371/journal.pone.0144599 (PMC4674124; doi:10.1371/journal.pone.0144599)
Supplement: S1 Table — Changes of serum FT4 concentrations during initial 6 months of anti-thyroid drug therapies (black circle, methimazole; white circle, propylthiouracil) from representative patients were demonstrated. (A) Stimulating activity-matched control group, and (B) blocking activity group. Stimulating activity, patients with thyroid-stimulating activity alone; blocking activity, patients with thyroid-stimulating activity combined with blocking activity; stimulating activity-matched control, patients from stimulating activity group who had matched values of initial free T4 and TBII to blocking activity group. (DOCX) [file pone.0144599.s002.docx]

|  | TBII (%) | Free T4 (ng/dL) | | Initial anti-thyroid treatment | | | Spontaneous hypothyroidism |
| --- | --- | --- | --- | --- | --- | --- | --- |
|  |  | baseline | 6mo | Drug type | Dosage (mg) | |  |
|  |  |  |  |  | Initial | 6mo |  |
| #1 | 94.0 | 2.11 | 0.75 | MMI | 15 | 5.0 | Yes |
| #2 | 71.2 | 2.23 | 0.85 | MMI | 20 | 5.0 | Yes |
| #3 | 85.8 | 2.78 | 0.79 | PTU | 300 | 50.0 | Yes |
| #4 | 75.8 | 2.29 | 0.81 | MMI | 20 | 5.0 | Yes |
| #5 | 97.9 | 2.23 | 0.42 | MMI | 20 | 2.5 | Yes |
| #6 | 98.0 | 2.88 | 0.95 | MMI | 30 | 2.5 | Yes |
| #7 | 57.7 | 2.39 | 0.39 | PTU | 300 | 50.0 | Yes |
| #8 | 92.1 | 2.43 | 0.55 | MMI | 20 | 2.5 | Yes |
| #9 | 91.4 | 2.78 | 0.92 | MMI | 30 | 5.0 | Yes |
| #10 | 54.9 | 2.19 | 0.60 | MMI | 20 | 5.0 | Yes |

S1 Table. Clinical characteristics of individual patients with blocking activity group (n=10).

MMI, methimazole; PTU, propylthiouracil.
